# Anti-nanodisc antibodies specifically capture nanodiscs and facilitate molecular interaction kinetics studies for membrane protein

**DOI:** 10.1038/s41598-023-38547-2

**Published:** 2023-07-19

**Authors:** Fuhito Nakagawa, Marin Kikkawa, Sisi Chen, Yasuomi Miyashita, Norie Hamaguchi-Suzuki, Minami Shibuya, Soichi Yamashita, Lisa Nagase, Satoshi Yasuda, Mitsunori Shiroishi, Toshiya Senda, Keisuke Ito, Takeshi Murata, Satoshi Ogasawara

**Affiliations:** 1 Department of Chemistry, Graduate School of Science, Chiba University, 1-33 Yayoi-Cho, Inage, Chiba 263-8522 Japan; 2 Department of Food and Nutritional Sciences, Graduate School of Integrated Pharmaceutical and Nutritional Sciences, University of Shizuoka, 52-1 Yada, Suruga-Ku, Shizuoka, 422-8526 Japan; 3 Membrane Protein Research Center, Chiba University, 1-33 Yayoi-Cho, Inage, Chiba 263-8522 Japan; 4 Structure Biology Research Center, Institute of Materials Structure Science, High Energy Accelerator Research Organization (KEK), 1-1 Oho, Tsukuba, Ibaraki 305-0801 Japan; 5 Department of Quantum Life Science, Graduate School of Science, Chiba University, 1-33 Yayoi-Cho, Inage, Chiba 263-8522 Japan; 6 Department of Biological Science and Technology, Tokyo University of Science, 6-3-1 Niijuku, Katsushika-Ku, Tokyo, 125-8585 Japan; 7 Department of Materials Structure Science, School of High Energy Accelerator Science, The Graduate University of Advanced Studies (Soken-Dai), 1-1 Oho, Tsukuba, Ibaraki 305-0801 Japan; 8 Faculty of Pure and Applied Sciences, University of Tsukuba, 1-1-1 Tennodai, Tsukuba, Ibaraki 305-8572 Japan; 9 Institute for Advanced Academic Research, Chiba University, 1-33 Yayoi-Cho, Inage, Chiba 263-8522 Japan

**Keywords:** Biological techniques, Analytical biochemistry, Biochemical assays, Biotechnology, Assay systems

## Abstract

Nanodisc technology has dramatically advanced the analysis of molecular interactions for membrane proteins. A nanodisc is designed as a vehicle for membrane proteins that provide a native-like phospholipid environment and better thermostability in a detergent-free buffer. This enables the determination of the thermodynamic and kinetic parameters of small molecule binding by surface plasmon resonance. In this study, we generated a nanodisc specific anti-MSP (membrane scaffold protein) monoclonal antibody biND5 for molecular interaction analysis of nanodiscs. The antibody, biND5 bound to various types of nanodiscs with sub-nanomolar to nanomolar affinity. Epitope mapping analysis revealed specific recognition of 8 amino acid residues in the exposed helix-4 structure of MSP. Further, we performed kinetics binding analysis between adenosine A_2a_ receptor reconstituted nanodiscs and small molecule antagonist ZM241385 using biND5 immobilized sensor chips. These results show that biND5 facilitates the molecular interaction kinetics analysis of membrane proteins substituted in nanodiscs.

## Introduction

Membrane proteins play key roles in various physiological processes, such as signal transduction, substance transport, enzyme catalysis, synaptic transmission regulation, and immune response, and are therefore, targets of more than 50% of therapeutic drugs^1^. Since most of these physiological processes involve the interaction of target proteins with small molecule ligands, the analysis of interactions between ligand and membrane proteins and screening of ligand libraries is crucial in drug discovery research. Surface plasmon resonance (SPR) is a well-established method for label-free evaluation of protein–ligand interactions in real-time with kinetics parameters such as association rate constants (k_on_) and dissociation rate constants (k_off_)^2,3^.

Membrane proteins are extracted from the cell membrane using detergents that are also used in subsequent purification and analysis processes. However, purification using detergents creates a highly dynamic environment wherein there is constant exchange of the detergent micelles surrounding the membrane proteins with those in the buffer. Thus, study protocols that require detergents may not provide a sufficiently stable environment for intermolecular interaction analysis, especially that of small molecule ligands^4–7^. Nanodiscs are an attractive alternative for solubilizing membrane proteins and have the added advantages of maintaining the lipid bilayer environment and improving thermal stability.

**Figure 1:**
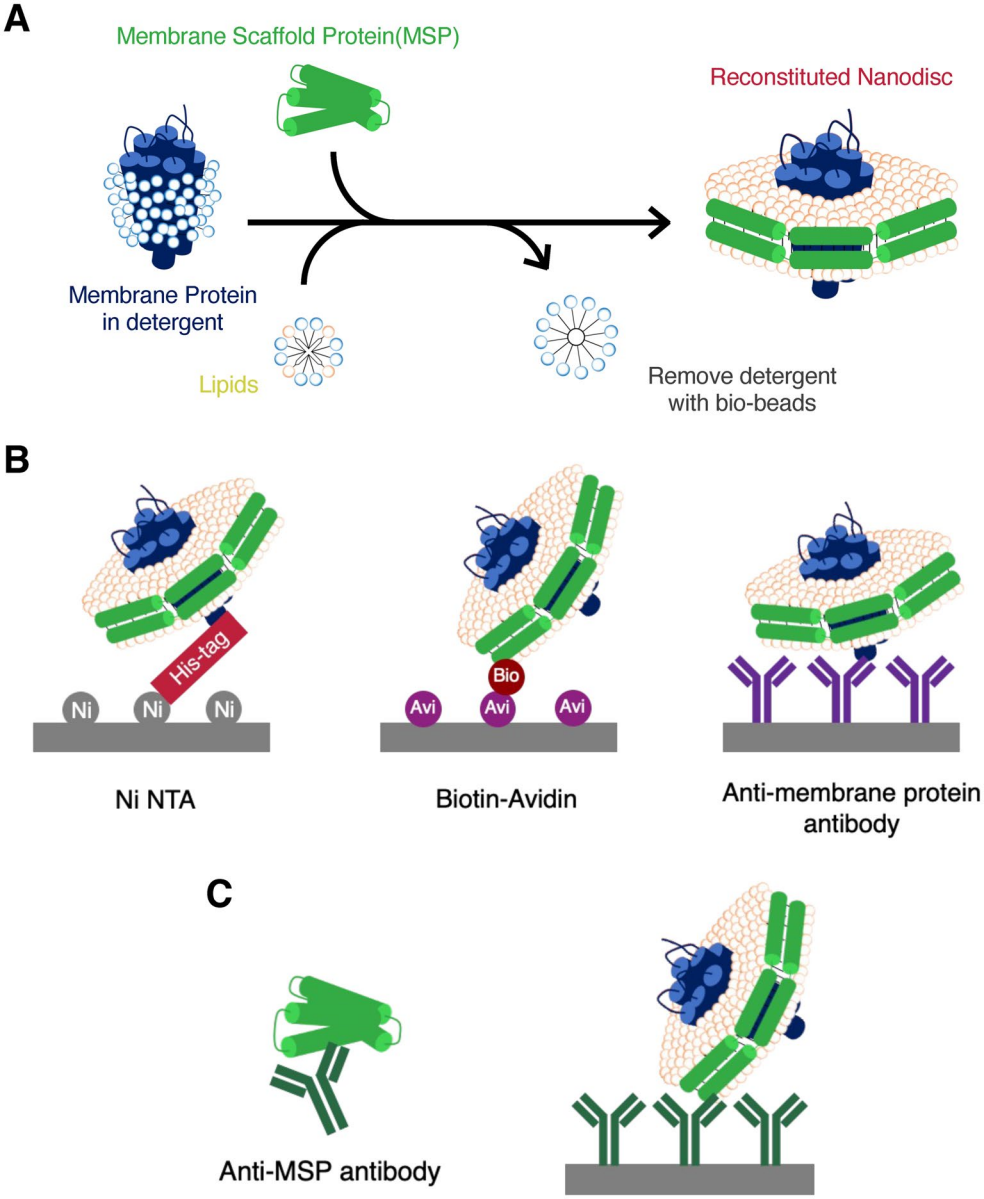
Nanodisc reconstruction and immobilization on an analysis chip using antibodies that capture nanodiscs. (**A**) General strategy for nanodisc reconstruction. Membrane proteins in detergents were mixed with MSP and lipids, and nanodiscs were reconstructed by removing detergents. (**B**) Three types of traditional nanodisc immobilization on an analysis chip. Immobilization methods using metal affinity, biotin–avidin complex, and anti-membrane protein antibody have been proposed, but each method has disadvantages. (**C**) Antibody against MSP and immobilization using anti-MSP antibodies. Capturing an analysis chip with an antibody that captures various types of nanodiscs is easy, fast, and efficient.

A nanodisc is one format of membrane protein, composed of discoidal lipid bilayers of phospholipids surrounded by amphipathic molecules, such as a membrane scaffold proteins (MSPs) (Fig. 1A)^8–11^. Since nanodiscs are without detergents and both the inside and outside regions of the membrane protein are exposed to aqueous solution, it is possible to analyze the interactions of ligands in solution, such as membrane-associating proteins, with the surface of the lipid bilayer. So nanodisc technology allows for more accurate kinetics analysis between membrane proteins and small molecule ligands using SPR^12^.

Kinetics analysis using SPR requires the immobilization of nanodiscs on the surface of a sensor chip (Fig. 1B). Typical immobilizations are performed using tag-mediated techniques, such as metal affinity between His-tagged nanodiscs and NTA chips, which allows characterization of binding constants of G-protein coupled receptors and small molecule antagonists^12–14^. However, these require a high level of capture that regulate non-specific binding owing to chip surface-exposed metal chelating residues^15^. A more stable immobilization is provided by biotin–avidin (Streptavidin) interaction using biotinylation of avi-tagged nanodiscs and SA chips, which is however, a cost-prohibitive method due to the difficulty of chip regeneration and the requirement of new chips for each experiment^16–18^.

Immobilization using antibodies against the membrane protein contained in nanodiscs is an efficient approach in terms of specificity for nanodiscs, and an unnecessity for tag attachment and chip reusability, but lacks versatility because of the necessity to generate antibodies with high affinity for each target membrane protein^12,19^. In contrast, antibodies against nanodiscs, corresponding to anti-MSP antibodies, can capture various nanodiscs directly on the sensor chip regardless of the membrane proteins, but this technique has not yet been developed (Fig. 1C).

Here, we report the development of anti-nanodisc antibodies to facilitate molecular interaction kinetics analysis for nanodisc studies. Our results suggest that anti-nanodisc antibodies are tag-free, versatile, and regenerable SPR analysis systems for various nanodiscs. These antibodies would be useful for drug screening and molecular interaction analysis of membrane protein studies in the future.

## Results

### Generation of monoclonal antibodies against MSP

To develop novel anti-MSP monoclonal antibodies, we immunized MRL/lpr mice with avi-tagged MSP1D1. The culture supernatants were screened using enzyme-linked immunosorbent assay (ELISA) for binding to purified as isolated avi-tagged MSP1D1. After performing a limiting dilution of the hybridomas, seven clones were established. The seven monoclonal antibodies were purified from culture supernatants using affinity purification with protein G. Of the seven monoclonal antibodies, six were confirmed to be reactive against the antigen by western blotting (Supplementary Fig. 1).

### Antibody epitope mapping on MSP1D1 using peptide arrays

**Figure 2:**
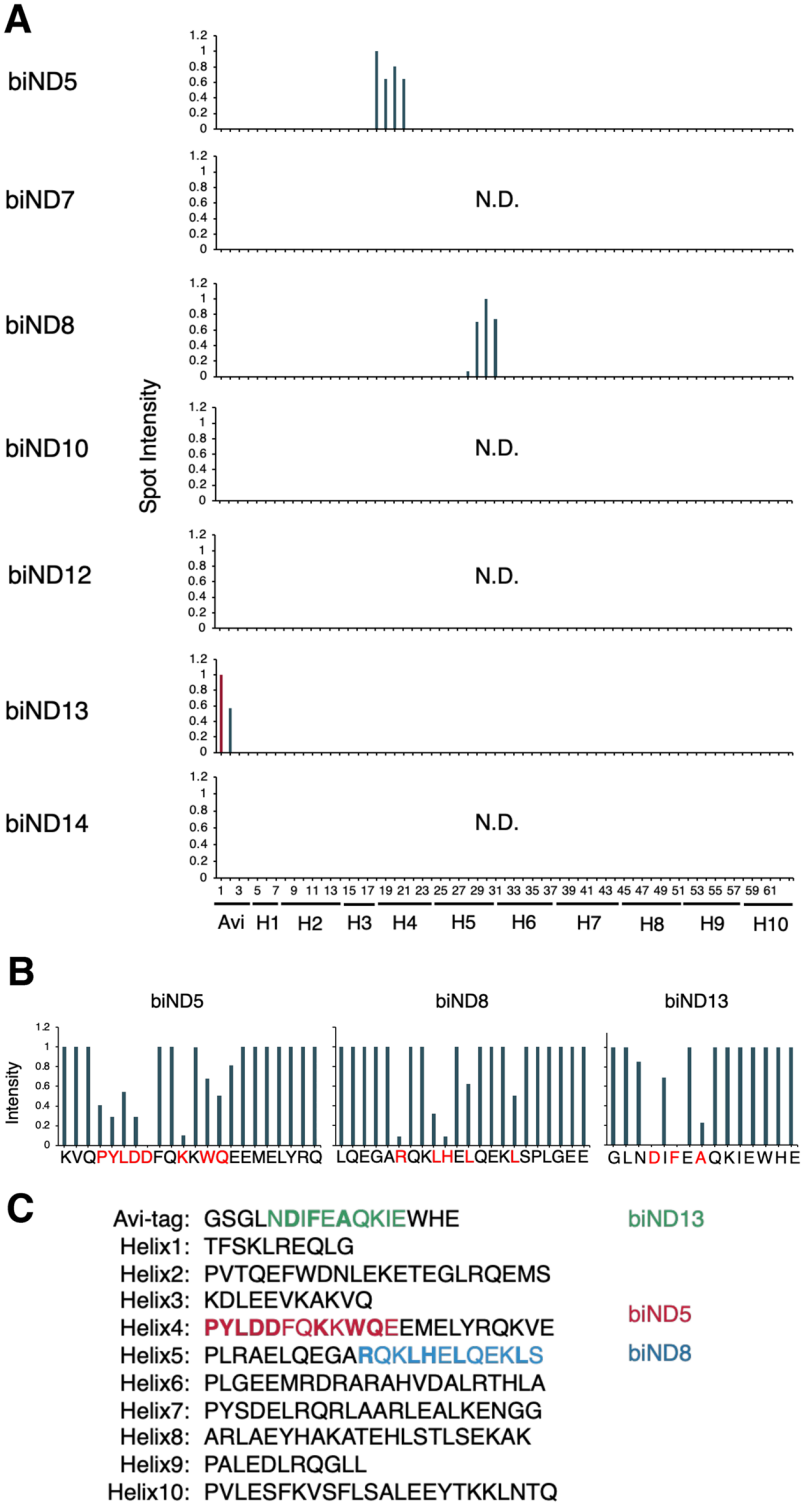
Avi-tagged MSP1D1 epitope mapping of seven monoclonal antibodies. To clarify the sequence recognized by the antibodies, the reactivity of the truncated and mutated peptides was tested. (**A**) The seven antibody epitopes identified with peptide arrays are shown on the respective avi-tagged MSP1D1 sequence. The spot intensity of the immunoreactivities is indicated by the line plots. (**B**) Alanine scanning analysis revealed amino acid residues that are suggested to be strongly involved in antibody-antigen binding because the spot intensity was significantly reduced owing to the mutations (shown in red). (**C**) The epitope of each antibody clone is shown in color: biND5 in red, biND8 in blue, and biND13 in green. Amino acid residues important for binding are shown in bold.

The epitopes of the seven monoclonal antibodies were analyzed using peptide arrays with spots consisting of 22 overlapping amino acid residues derived from avi-tagged MSP1D1 as antigen. Using this epitope mapping with the array, we successfully identified three (biND5, biND8, and biND13) out of seven antibody epitope regions on avi-tagged MSP1D1. It was found that biND5 bound to the first part of helix 4 region included in the MSP sequence; biND8 also recognized the MSP sequence, but its epitope was contained in the latter half area of helix 5 region; whereas biND13 recognized an amino acid residue region containing an avi-tag sequence, and was therefore considered an anti-avi-tag antibody (Fig. 2A).

To determine the contribution of each amino acid residue to antigen binding, we synthesized peptides containing point mutations to alanine or leucine. Peptides were synthesized based on the 22-amino acid residue peptides with high spot intensity in previous experiments on biND5 and biND8, and the 15-amino acid residue avi-tag sequence for biND13. Based on alanine scanning epitope analysis, we found that biND5 strongly recognized the helix 4 region which covers twelve amino acid residues, biND8 bound to five amino acid residues included in helix 5, and biND13 recognized the three amino acid residues contained in the avi-tag sequence (Fig. 2B).

Furthermore, to investigate the suitable peptide length of antigens, we prepared spot strips consisting of truncated peptides with three sequential amino acid deletions from the 22 amino acid peptide and examined the reactivities using shorter-length peptide array spots. As expected, sufficient reactivity of each antibody against the peptide strips was preserved at the spots with 10 to 15 amino acid residues containing the highly contributing amino acids (Supplementary Fig. 2).

This epitope mapping analysis identified two anti-MSP antibodies, biND5 and biND8, containing different epitopes, and one anti-avi-tag antibody, biND13 (Fig. 2C).

### Affinity measurement of the anti-MSP antibodies

**Figure 3:**
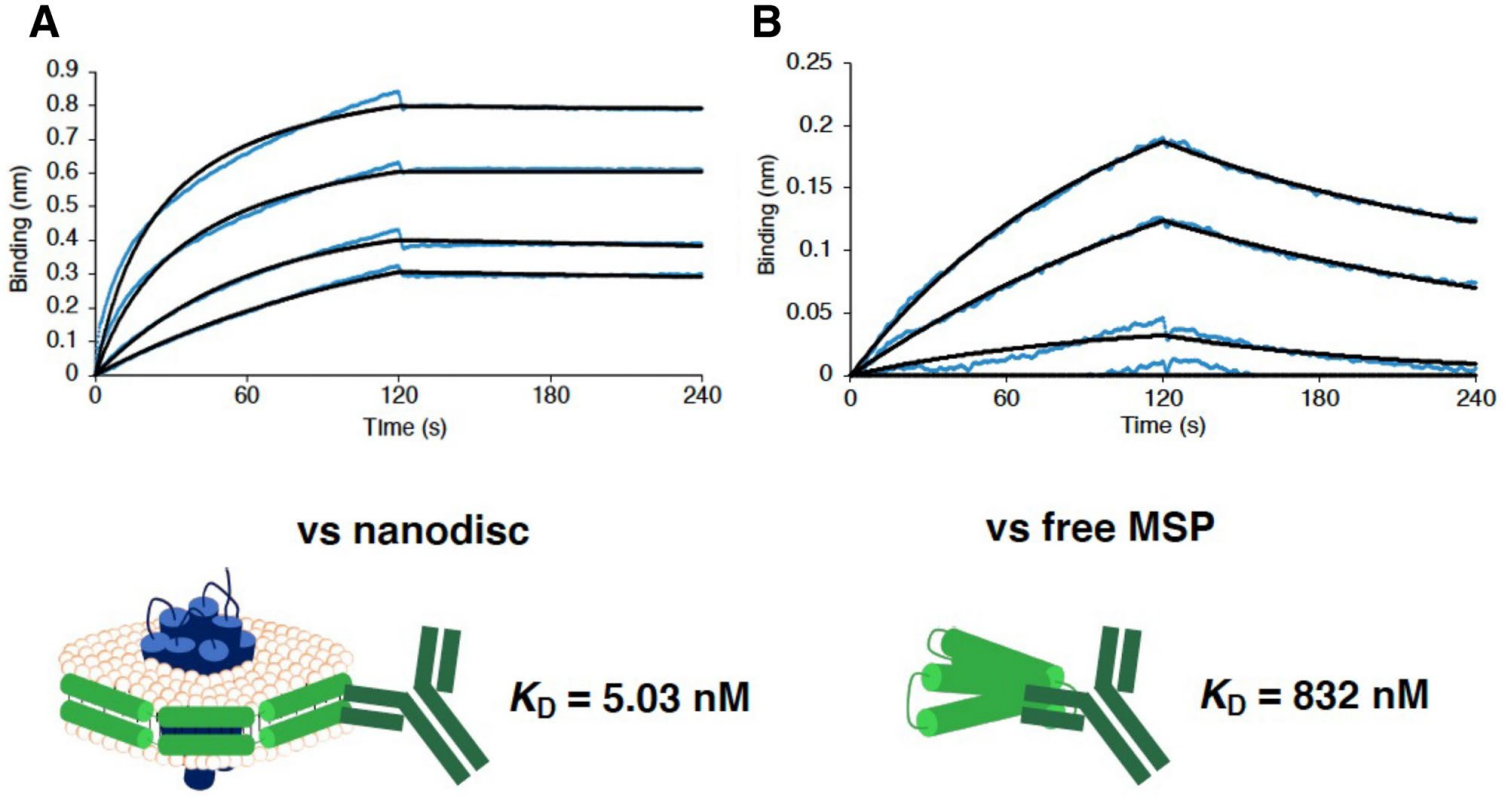
Affinity against nanodisc (MSP1D1) and free MSP1D1. BLI sensorgrams between biND5 immobilized on biosensor and nanodisc reconstituted with MSP1D1 (1000–500–250–100 nM; **A**) or free MSP1D1 (1000–500–250–100 nM; **B**). The data are depicted in blue, and the fit data to a 1:1 binding model are shown in black.

To assess the kinetics parameters of all seven antibodies against free MSPs and nanodiscs, we prepared four types of MSPs (MSP1, MSP1D1, MSP1E3D1, and MSP2N2) and four types of thermophilic rhodopsin (TR) nanodiscs were reconstituted in each MSP. Thereafter, we tested their affinity using bio-layer interferometry (BLI). Each antibody was immobilized on the biosensor, and target proteins at various concentrations were added to determine the kinetics parameters based on global fitting. The seven clones exhibited a micromolar-level affinity against free MSPs, but all antibodies showed significantly higher affinity against reconstituted nanodiscs than free MSPs (Fig. 3, Table 1, and Supplementary Tables 1–3). Notably, one of the anti-MSP antibodies, biND5, bound to all types of TR nanodiscs with sub-nanomolar to nanomolar affinity (*K*_D_ = 0.78–5.0 nM) and exhibited the lowest off rate amongst established antibodies. biND5 recognized 8 amino acid residues in the exposed helix-4 structure of MSP sequences (Fig. 3B) and these amino acids were biased to one side of the helix4. Accordingly, biND5 showed high affinity and specificity for nanodiscs thus qualifying as an anti-nanodisc antibody.

**Table 1:** Affinity against nanodisc (MSP1D1) and free MSP1D1.

Clones	Nanodisc (MSP1D1)			Free MSP1D1		
	*K*_D_ (nM)	*k*_on_ (1/Ms) × 10^4^	*k*_off_ (1/s) × 10^−4^	*K*_D_ (μM)	*k*_on_ (1/Ms) × 10^4^	*k*_off_ (1/s) × 10^−4^
**biND5**	**5.03**	**4.27**	**2.05**	**0.832**	**0.572**	**25.2**
biND7	16.7	4.83	8.04	4.10	1.66	681
biND8	71.1	5.56	39.6	3.39	0.882	299
biND10	16.2	2.59	4.20	17.5	0.207	362
biND12	63.9	0.706	4.51	729.0	0.00480	350
biND13	52.7	1.19	6.27	19.2	0.0797	153
biND14	96.4	2.41	23.2	635.0	0.00177	112

The biND5 (bold) was the strongest affinity in this study, so we proceeded with further experiments.

### Kinetic analysis for nanodisc with SPR using the anti-nanodisc antibody

**Figure 4:**
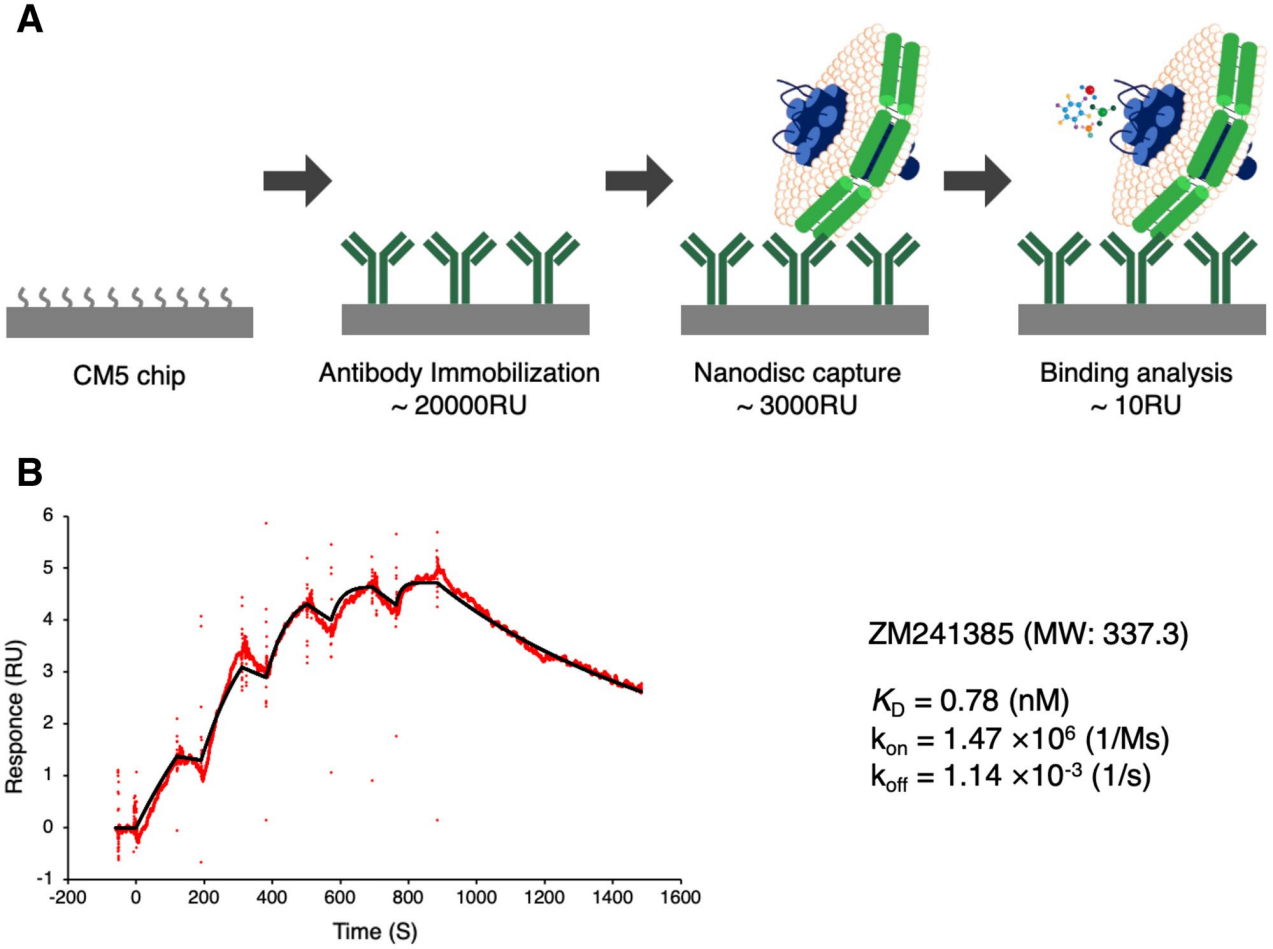
Kinetics analysis of ligands against nanodiscs using SPR. (**A**) Overview of the analysis system. Kinetics analysis was performed by immobilization of the antibody on the sensor chip, followed by nanodisc capture and injection of small molecules. (**B**) Response curves obtained with A2aR-nanodiscs captured by biND5 immobilized sensor chip as shown on a panel for ZM241385 (3.125–6.25–12.5–25–50 nM) and determined binding parameters by SPR. Data fit to a 1:1 binding model are shown in black.

Finally, we performed kinetic analysis of the molecular interactions between small molecule antagonist ZM241385 and adenosine A_2a_ receptor (A2aR)-reconstituted nanodiscs immobilized by anti-nanodisc antibody biND5 using SPR (Fig. 4A). biND5 was immobilized on the CM5 chip using typical amine coupling until 12,000 response units (RU), and then the A2aR-nanodisc was captured. Nanodiscs were captured with 1800 RU on the CM5 chip-immobilized biND5. Empty nanodiscs were captured in the same way on the reference flow cell until 1200 RU. Nanodiscs that dissociated from antibodies during the measurement were 10 to 15 RU per hour.

The binding constants were determined for small molecule antagonists using the single-cycle kinetics (SCK) method of SPR. After the blank (no analyte) cycle, analytes at five sequential concentrations were injected to calculate the kinetics parameters based on 1:1 binding fitting of sensorgrams subtracted from the blank. The kinetics analysis of ZM241385 the A2aR-nanodisc immobilized by biND5 was performed (Fig. 4B). The kinetics parameters determined are closely correlated with a previous study^20^. Similarly, kinetic analyses of the molecular interaction were able to measure between other ligands and A2aR-nanodisc (Supplement Fig. 3). The antibody-immobilized chip could be regenerated by injection of 100 mM glycine HCl pH 2.6, and biND5 maintained its binding activity to nanodiscs (Supplement Fig. 4).

## Discussion

Despite the central importance of SPR-based molecular interaction analysis of membrane proteins in drug discovery research, no technology has been developed yet to efficiently capture membrane proteins reconstituted in nanodisc on a chip for analysis. Antibodies against nanodiscs, corresponding to anti-MSP antibodies, can capture various nanodiscs directly on the sensor chip with high specificity, independent of the reconstituted membrane proteins, and without any extra modifications, such as biotinylation.

**Figure 5:**
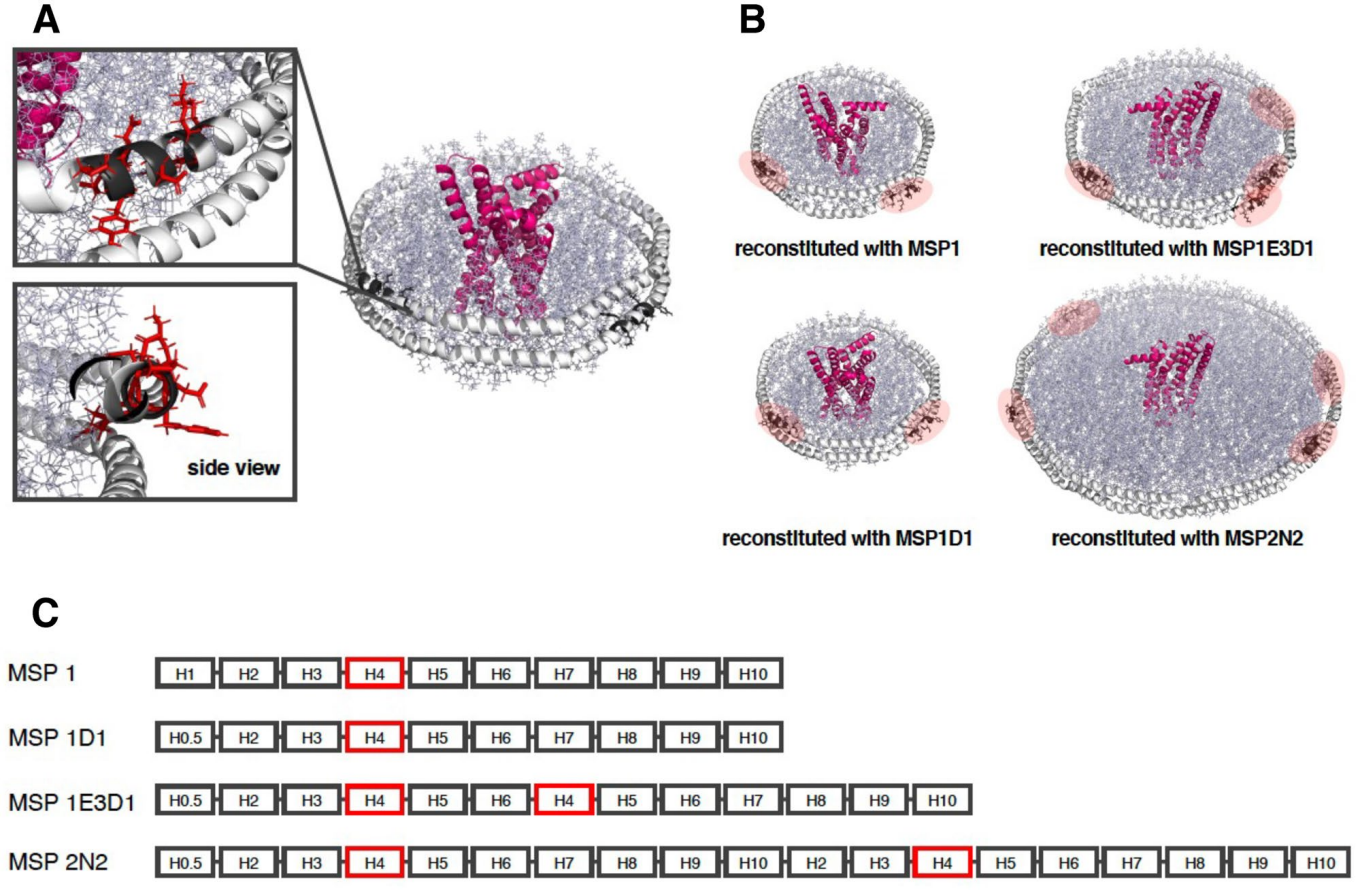
Binding site structure of the anti-nanodisc antibody; biND5. (**A**) Structural model of nanodisc reconstituted with MSP1D1. MSPs are shown in white, membrane protein in pink, lipids in blue-white. Amino acid residues on the MSP that are specifically recognized by biND5 are shown in black, and their exposed side chains are shown in red. (**B**) Structural model of nanodisc reconstituted with MSP1, 1D1, 1E3D1, and 2N2, respectively. The binding sites for biND5 on each MSP are highlighted in red; the nandisc reconstituted with MSP1 and 1D1 have two biND5 binding sites, and those reconstituted with MSP1E3D1 and 2N2 have four binding sites. (**C**) Sequences of the four MSP variants. Each variant is composed of a helical structure, and has repeating helix regions. Helices shown in red correspond to the binding sites for biND5.

In this study, we generated anti-nanodisc antibodies that specifically recognize reconstituted nanodiscs and thereafter, used these antibodies to develop an SPR analysis system. Many MSPs contain the His-tag for purification, but the His-tag was cut to raise purity by further purification step according to general method for reconstitution of nanodiscs. Accordingly, these antibodies were very useful for handling the nanodiscs. All the anti-MSP antibodies generated displayed a higher affinity for reconstituted nanodiscs than free MSPs. The fact that the nanodisc-specific antibodies could be generated even though the mice were immunized with free MSPs implies that MSPs have a nanodisc-like structure in mice. The crystal structure of Apolipoprotein-A1 (PDB ID: 1AV1), which was the basis of MSPs, was a tetrameric nanodisc-like structure^21^. It suggests that it is easy to take on a nanodisc-like state in solution. Therefore, several antibodies that fit the nanodisc structure and bind to it could be obtained. Although biND13 is an anti-avi-tag antibody, we consider that its higher affinity for nanodiscs is due to the fact that the avi-tag fused to the MSP is more easily exposed in nanodiscs than in solution. The epitope of biND5 based on the nanodisc reconstituted with MSP1D1 is shown in Fig. 5A. In this model, amino acid residues (shown in black) and their chains (shown in red) that biND5 specifically recognized on the MSP sequence are exposed on the outside of nanodisc structure. Thus, the nanodisc specificity of biND5 is assumed to be due to its higher accessibility and steric recognition of epitopes exposed on the outside of MSPs reconstituted in the nanodisc. Furthermore, these antibodies showed higher affinity for longer MSPs (1E3D1 and 2N2) than shorter MSPs (1 and 1D1). The longer MSP variants are formed by repeating their helices containing sequences to which the antibodies bind, and hence the increased number of antibody binding sites also resulted in higher affinity (Fig. 5B,C). Recently, several new membrane scaffold proteins (MSPs) have been developed^22–25^. MSPs of NW9, NW11, NW30 and NW50 have been developed from Apolipoprotein A-1^22–24^ including same amino acid sequence of biND5 epitope, might be able to bind these nanodiscs. But saponinA is different protein from Apolipoprotein A-1^25^, biND5 might be unable to bind the nanodisc reconstituted with saponinA. The strong binding of biND5 against the nanodisc formed as described above allowed for the stable capture of nanodiscs onto the analysis chip in SPR, and the subsequent analysis of small molecule binding.

The anti-nanodisc antibody; biND5 is the first reported antibody with nanodisc specific properties and can capture various types of nanodiscs with high affinity while retaining its binding activity of the antibody even after immobilization on an analysis chip by amine coupling or acid regeneration. This antibody is a tool that can capture nanodiscs with specificity, high affinity, and tag-free, and thus has potential applications in various studies, such as library screening and molecular interaction analysis that require the immobilization of membrane proteins. We believe that anti-nanodisc antibodies have the potential to promote and streamline drug discovery research on membrane proteins.

## Materials and methods

### Sample preparation

#### MSPs

Membrane scaffold proteins MSP1, MSP1D1, MSP1E3D1, and MSP2N2, which contain 7 × His-tag, a 3C protease cleavage site (LEVLFQGP), and an avi-tag (GLNDIFEAQKIEWH) at the N-terminus, were expressed in *Escherichia coli* BL21(DE3) RIL using a pET28 vector. The bacteria were grown at 37 °C in TB medium supplemented with 50 μg/mL kanamycin. The cultures were grown to an OD600 of 2.5–3.0 prior to induction with 0.5 mM isopropyl β-d-1-thiogalactopyranoside (Wako, Osaka, Japan). The bacterial cells were harvested for 2–3 h following induction, and the bacterial pellet was stored at − 80 °C until further processing. Cell pellets were resuspended in a solution containing 40 mM Tris–HCl pH 8.0, 300 mM NaCl, 1% Triton X-100 (Sigma-Aldrich, St. Louis, MO, USA), and 1 mM Phenylmethylsulfonyl fluoride (Nacalai Tesque, Kyoto, Japan) and then lysed by sonication. Following centrifugation at 18,000*g* for 30 min at 4 °C, the supernatant was loaded onto a Ni Sepharose 6 Fast Flow column. The column was washed with 10 column volumes (CV) of a solution containing 40 mM Tris–HCl (pH 8.0), 300 mM NaCl, and 1% Triton X-100 followed by a second wash of a solution containing 10 CV of 40 mM Tris–HCl pH 8.0, 300 mM NaCl, 50 mM Na-Cholate, and 10 mM imidazole, and a third wash of a solution containing 40 mM Tris–HCl pH 8.0, 300 mM NaCl, 0.05% *n*-dodecyl β-d-maltoside (DDM; Anatrace, Maumee, OH, USA), and 20 mM imidazole. The target protein was eluted with a solution containing 40 mM Tris–HCl pH 8.0, 300 mM NaCl, 0.05% DDM, and 100 mM imidazole. The eluate was concentrated with Amicon Ultra 10K (Merck Millipore, Burlington, MA, USA), and the buffer exchanged with a solution containing 40 mM Tris–HCl (pH 8.0), 300 mM NaCl, and 0.05% DDM. The N-terminal His-tag was cleaved with 3C protease during an overnight incubation at 4 °C for 16 h, followed by the removal of the uncleaved target and protease with a second Ni affinity step, wherein the flow-thorough was collected.

#### TR nanodiscs

Expression and purification of thermophilic rhodopsin was performed following previously published methods^26^. Purified MSP was added at a molar ratio of 1:10:350 (TR:MSP:lipid) to the detergent-solubilized TR sample containing *E. coli* polar lipid extracts. The mixtures were incubated at 4 °C for 1 h, following which the detergent was removed using Bio-Beads SM-2 (Bio-Rad, Hercules, CA, USA). The solubilized nanodiscs were isolated by centrifugation at 150,000*g* for 30 min. Thereafter, the supernatant was loaded onto an Ni affinity chromatography column to separate TR containing nanodiscs from the excess empty nanodiscs. The Ni column eluate was subsequently concentrated with Amicon Ultra 30K (Merck Millipore, Burlington, MA, USA).

#### A2aR (wt)-nanodiscs

Wild type A2aR DNA was synthesized and cloned into a modified pHEK293 Ultra Expression Vector I (TaKaRa Bio Inc., Shiga, Japan) containing a green fluorescent protein followed by an HRV3C (human rhinovirus 3C) protease site (LEVLFQ/GP) and an 8 × His-tag at the C terminus. Expi293F (Thermo Fisher Scientific, MA, USA) cells were grown and maintained in HE200 CD medium (Gmep Inc., Fukuoka, Japan) supplemented with 4 mM l-alanyl-l-glutamine (Nacalai Tesque) and a mixture of penicillin, streptomycin, and amphotericin B (Nacalai Tesque) in an agitator at 130 rpm in a humidified, 8% CO_2_ incubator at 37 °C. Transfection with Polyethylenimine (PEI) was carried out at a density of 3 × 10^6^ cells/mL cells. Thereafter, the cells were collected and solubilized in a solution containing 20 mM HEPES pH 7.5, 300 mM NaCl, 1% DDM, and 0.2% cholesteryl hemisuccinate (CHS; Sigma-Aldrich, Saint Louis, MO, USA). The sample was stirred at 4 °C for 2 h, following which the insoluble fraction was discarded by centrifugation at 15,000*g* for 15 min. Anti-His-tag monoclonal antibody-immobilized resin was prepared using NHS-activated sepharose (Cytiva, Marlborough, MA, USA) to isolate the 8 × His-tagged A2aR from the soluble fraction. The resin was washed in a solution containing 20 mM HEPES pH 7.5, 150 mM NaCl, 0.03% DDM, and 0.006% CHS. Purified MSP1D1 and 1-palmitoyl-2-oleoyl-sn-glycero-3-phosphocholine (POPC) were added to the A2aR-immobilized resin at a molar ratio of 1:10:500 (A2aR:MSP:POPC). The mixtures were incubated at 4 °C for 1 h and the detergent was then removed using Bio-Beads. Thereafter, following the removal of Bio-Beads, the resin was washed in a solution containing 20 mM HEPES pH 7.5 and 150 mM NaCl. HRV3C protease was used to cleave A2aR containing nanodiscs from the antibody-immobilized resin. Subsequently, the eluate was centrifuged at 150,000*g* for 30 min at 4 °C, and the supernatant was concentrated with Amicon Ultra 30K. The sample was loaded onto a Superdex 200 Increase 10/300 GL column (GE Healthcare, Chicago, IL, USA) equilibrated in a solution containing 20 mM HEPES pH 7.5 and 150 mM NaCl. Peak fractions were collected, concentrated with Amicon Ultra 30K, and stored at − 80 °C until further use.

#### Empty nanodiscs

To assemble empty nanodiscs, MSPs and lipids specific to each target nanodisc were mixed, and the mixture rotated at 4 °C for 1 h. This was followed by the addition of bio-beads and rotation of the sample at 4 °C for 16 h to remove the detergent. The solubilized nanodiscs were then isolated by centrifugation at 150,000 g for 30 min. The supernatant was concentrated with Amicon Ultra 10K and injected into a Superdex 200 Increase 10/300 GL column equilibrated in 20 mM HEPES pH 7.5 and 150 mM NaCl. Fractions containing empty nanodiscs were eluted first and confirmed by SDS-PAGE.

### Experimental animals

4-week-old female MRL/MpJJmsSlc-*lpr/lpr* mice were purchased from Nihon SLC (Shizuoka, Japan). The temperature of the animal room was controlled at 20–25 °C, and the lighting was alternated between day and night at 12 h/12 h. Procedures described in this study conformed to the guidelines outlined in the Guide for the Care and Use of Laboratory Animals of Japan and were approved by the Chiba University Animal Care Committee (approval number 2-55), and performed according to ARRIVE guidelines.

### Hybridoma production and IgG purification

Two 4-week-old female MRL/MpJJmsSlc-*lpr/lpr* mice were immunized by intraperitoneal (i.p.) injection of 100 μg purified MSP1D1 protein along with Imject Alum (Thermo Fisher Scientific) every 10 days. A booster injection was administered i.p. 2 days prior to euthanizing the mice by cervical dislocation. Spleen cells were harvested and fused with P3X63Ag8.U1 (P3U1) (ATCC, Manassas, VA, USA) myeloma cells using PEG1500 (Roche Diagnostics, Indianapolis, IN, USA). The hybridomas were cultured in E-RDF medium (KYOKUTO PHARMACEUTICAL Co., Ltd., Tokyo, Japan) containing hypoxanthine, aminopterin, and thymidine, 10% (v/v) fetal bovine serum (Thermo Fisher Scientific), and 5% (v/v) BM supplemented with H1 (Roche Diagnostics). Culture supernatants were screened using enzyme-linked immunosorbent assay (ELISA). Purified MSP1D1 protein (1 μg/mL) was immobilized on Nunc Maxisorp 96-well plates (Thermo Fisher Scientific). After blocking with 1% bovine serum albumin (BSA) in 0.05% Tween20/phosphate-buffered saline (PBS; Nacalai Tesque), the plates were incubated with culture supernatants, following which peroxidase-conjugated anti-mouse IgG diluted 1:20,000 (Jackson ImmunoResearch, West Grove, PA, USA) was added. After each antibody reaction step, 10 times wash procedure was performed by 96-well plate washer (Immunowash 1575, Bio-rad). The enzymatic reaction was performed using a 1-Step Ultra TMB-ELISA (Thermo Fisher Scientific). Optical density was measured at 655 nm using an EPOCH microplate reader (US BioTek Laboratories, Shoreline, WA, USA). 10 strongest wells among ELISA positive wells were selected and tried to establish monoclonal cells. IgGs were affinity-purified from hybridoma culture supernatants using protein G Sepharose Fast Flow (Cytiva). Binding antibodies were first eluted with 10 mL 100 mM glycine–HCl pH 2.6 and then neutralized with 1 mL 1 M Tris–HCl (pH 9.0). Purified IgG was concentrated using Amicon Ultra 50K (Merck Millipore) and the buffer was replaced into PBS for further experiments.

### Peptide arrays

To prepare a peptide array sheet, array spots (each spot with a diameter of approximately 3 mm) consisting of amino acid residues derived from the sequence of avi-tagged MSP1D1 were synthesized on a cellulose membrane (IntavisAG, Bioanalytical Instruments, Nattermannalle, Germany) using a ResPep SL automatic peptide synthesizer (IntavisAG) based on previously reported methods^27^. The side-chain protecting groups were removed by treatment with trifluoroacetic acid after completion of peptide synthesis. Thereafter, the membrane was cut up for immunoblotting with the respective antibodies. After blocking with Blocking One (Nacalai Tesque) for 1 h at 22 °C, the membranes were incubated with the respective antibodies for 1 h at room temperature. Subsequently, after washing with PBS and 0.05% Tween 20 (PBST), the membranes were further incubated with horseradish peroxidase-conjugated anti-mouse IgG Fc fragment specific for 30 min at room temperature. Then, after washing with PBST, the membranes were developed using for EzWestBlue (ATTO, Tokyo, Japan) 10 min. Finally, following the reaction, the membranes were scanned, and the reactive and signal intensity of each spot was determined and analyzed using ImageJ software (https://imagej.nih.gov/ij/index.html).

### Bio-layer interferometry

Affinity measurements were performed against the seven monoclonal anti-MSP antibodies on an anti-mouse immunoglobulin G Fc capture biosensor (ForteBio, Fremont, CA, USA) using the BLItz system (ForteBio) following the manufacturer’s instructions. Binding assays were performed at 22 °C. For kinetic measurements of free MSPs, four concentrations (1000 nM, 500 nM, 200 nM, and 100 nM) in 40 mM Tris–HCl pH 8.0, 300 mM NaCl, and 0.05% DDM were used. For TR nanodiscs, four concentrations (500 nM, 200 nM, 100 nM, and 50 nM) in 50 mM Tris–HCl pH 7.5 and 1 M NaCl were used. Each step in the binding assays was as follows: equilibration, association, and dissociation for 120 s each. Kinetic constants were determined using BLItz software (BLItz Pro 1.2, ForteBio) according to global fitting of datasets.

### Surface plasmon resonance

The SCK method of SPR can determine the binding constants (kon, koff, and KD) quickly and easily by sequentially injecting a series of analyte concentration into the targets immobilized on the sensor chip in one cycle with no regeneration between sample injection. However, it requires optimizations of experimental conditions (dilution factor, maximum concentration, and association and dissociation time)^28–30^.

#### Antibody immobilization

Antibody immobilization was performed at 25 °C on a Biacore T200 instrument (GE Healthcare) using a solution containing 20 mM HEPES pH 7.5 and 150 mM NaCl as running buffer. Anti-nanodisc antibodies were immobilized onto the CM5 sensor chip (Cytiva) by injecting a solution containing 100 μg/mL biND5 pH 5.0 at a flow rate of 10 μL/min through standard amine coupling, reaching levels at 12,000 RU.

#### Kinetics analysis of A2aR-nanodiscs

Kinetics analysis was performed at 15 °C using a solution containing 20 mM HEPES pH 7.5, 150 mM NaCl, and 1% dimethyl sulfoxide as running buffer. A2aR nanodiscs were captured onto the active flow cell of the biND5 immobilized chip at 1800 RU. Empty nanodiscs for A2a nanodiscs were captured onto the reference flow cell to reach levels of 1200 RU, considering the mass difference. Kinetics analysis between nanodiscs and ZM241385 were performed using a single-cycle kinetics method. After the startup, which was followed by the solvent correction cycle and dummy cycles, analytes were injected at increasing concentrations. Analyte responses were corrected for buffer responses and subtracted for reference and blank responses. Curve fitting and data analysis were performed using the Biacore T200 Evaluation software (GE Healthcare).

### A2aR-nanodisc model building

The Nanodisc model was created by the nanodisc builder in the CHARMM-GUI program^31,32^. The A2aR structure was constructed with reference to PDB ID 3VG9.

## Supplementary Information

Supplementary Information.

## Data Availability

The datasets generated and/or analysed during this study are available from the corresponding author on reasonable request.

## References

[1] Hopkins AL, Groom CR (2002). The druggable genome. Nat. Rev. Drug Discov..

[2] Rich RL, Myszka DG (2000). Advances in surface plasmon resonance biosensor analysis. Curr. Opin. Biotechnol..

[3] McDonnell JM (2001). Surface plasmon resonance: Towards an understanding of the mechanisms of biological molecular recognition. Curr. Opin. Chem. Biol..

[4] Harding PJ, Hadingham TC, McDonnell JM, Watts A (2006). Direct analysis of a GPCR–agonist interaction by surface plasmon resonance. Eur. Biophys. J..

[5] Zhukov A (2011). Biophysical mapping of the adenosine A2A receptor. J. Med. Chem..

[6] Aristotelous T (2013). Discovery of β2 adrenergic receptor ligands using biosensor fragment screening of tagged wild-type receptor. ACS Med. Chem. Lett..

[7] Navratilova I, Dioszegi M, Myszka DG (2006). Analyzing ligand and small molecule binding activity of solubilized GPCRs using biosensor technology. Anal. Biochem..

[8] Bayburt TH, Sligar SG (2010). Membrane protein assembly into nanodiscs. FEBS Lett..

[9] Denisov IG, Sligar SG (2017). Nanodiscs in membrane biochemistry and biophysics. Chem. Rev..

[10] Denisov IG, Sligar SG (2016). Nanodiscs for structural and functional studies of membrane proteins. Nat. Struct. Mol. Biol..

[11] Rouck JE (2017). Recent advances in nanodisc technology for membrane protein studies (2012–2017). FEBS Lett..

[12] Bocquet N (2015). Real-time monitoring of binding events on a thermostabilized human A2A receptor embedded in a lipid bilayer by surface plasmon resonance. Biochim. Biophys. Acta.

[13] Rich RL, Errey J, Marshall F, Myszka DG (2011). Biacore analysis with stabilized G-protein-coupled receptors. Anal. Biochem..

[14] Trahey M (2015). Applications of lipid nanodiscs for the study of membrane proteins by surface plasmon resonance. Curr. Protoc. Protein Sci..

[15] Francis S, Willard FS, Siderovski DP (2006). Covalent immobilization of histidine-tagged proteins for surface plasmon resonance. Anal. Biochem..

[16] Yoshida K (2019). Phospholipid membrane fluidity alters ligand binding activity of a G protein-coupled receptor by shifting the conformational equilibrium. Biochemistry.

[17] Xu H (2015). Characterization of the direct interaction between KcsA-Kv1.3 and its inhibitors. Biochim. Biophys. Acta.

[18] Sharma S, Wilkens S (2017). Biolayer interferometry of lipid nanodisc-reconstituted yeast vacuolar H+-ATPase. Protein Sci..

[19] Ritchie TK, Kwon H, Atkins WM (2011). Conformational analysis of human ATP-binding cassette transporter ABCB1 in lipid nanodiscs and inhibition by the antibodies MRK16 and UIC2. J. Biol. Chem..

[20] Hino T (2012). G-protein-coupled receptor inactivation by an allosteric inverse-agonist antibody. Nature.

[21] Borhani DW, Rogers DP, Engler JA, Brouillette CG (1997). Crystal structure of truncated human apolipoprotein A-I suggests a lipid-bound conformation. Proc. Natl. Acad. Sci. U.S.A..

[22] Nasr ML (2017). Covalently circularized nanodiscs for studying membrane proteins and viral entry. Nat. Methods.

[23] Zhang S (2021). One-step construction of circularized nanodiscs using SpyCatcher-SpyTag. Nat. Commun..

[24] Ren Q, Zhang S, Bao H (2022). Circularized fluorescent nanodiscs for probing protein–lipid interactions. Commun. Biol..

[25] Frauenfeld J (2016). A saposin-lipoprotein nanoparticle system for membrane proteins. Nat. Commun..

[26] Akiyama T (2021). Further thermo-stabilization of thermophilic rhodopsin from *Thermus thermophilus* JL-18 through engineering in extramembrane regions. Proteins.

[27] Su H (2019). Insight of diagnostic performance using B-cell epitope antigens derived from triple P44-related proteins of *Anaplasma*
*phagocytophilum*. Diagn. Microbiol. Infect. Dis..

[28] Kamat V, Rafique A (2017). Extending the throughput of Biacore 4000 biosensor to accelerate kinetic analysis of antibody–antigen interaction. Anal. Biochem..

[29] Palau W, Primo CD (2013). Simulated single-cycle kinetics improves the design of surface plasmon resonance assays. Talanta.

[30] Palau W, Primo CD (2012). Single-cycle kinetic analysis of ternary DNA complexes by surface plasmon resonance on a decaying surface. Biochimie.

[31] Jo S, Kim T, Iyer VG, Im W (2008). CHARMM-GUI: A web-based graphical user interface for CHARMM. J. Comput. Chem..

[32] Qi Y, Lee Y, Klauda JB, Im W (2019). CHARMM-GUI nanodisc builder for modeling and simulation of various nanodisc systems. J. Comput. Chem..

