# Supplementary material for: Anti-nanodisc antibodies specifically capture nanodiscs and facilitate molecular interaction kinetics studies for membrane protein
Source: Sci Rep. 2023 Jul 19;13:11627. doi: 10.1038/s41598-023-38547-2 (PMC10356912; doi:10.1038/s41598-023-38547-2)
Supplement: Supplementary file 1 — Supplementary Information. [file 41598_2023_38547_MOESM1_ESM.pdf]

Supplementary Figures Legend

Supplementary Figure S4. Western blotting analyses of established antibodies in this study.

Lane1: MSP1, 2: MSP1D1, 3: MSP1E3D1, 4: MSP2N2, and 5: Avi-tag fused protein were applied.

Supplementary Figure S2. The minimum epitope of biND5, biND8, and biND13.

As a results of the 22-amino acid residue peptides array using by biND5, biND8, and biND13, shorter peptides were synthesized and analyzed.

Supplementary Figure S3. Kinetics analysis of ligands against A2aR-nanodiscs using SPR.

Response curves obtained with A2aR-nanodiscs captured by biND5 immobilized sensor chip as shown on a panel for Etrumadenant (A) and XAC (B), and determined binding parameters by SPR. Data fit to a 1:1 binding model are shown in black.

Supplementary Figure S4. Full sensorgrams kinetics analysis of ligands against A2aR-nanodisc using biND5 immobilized sensor chip.

Starting points of each step were indicated by arrows. a: A2aR-nanodisc capture, b: association step, c: dissociation step, and d: regeneration step.

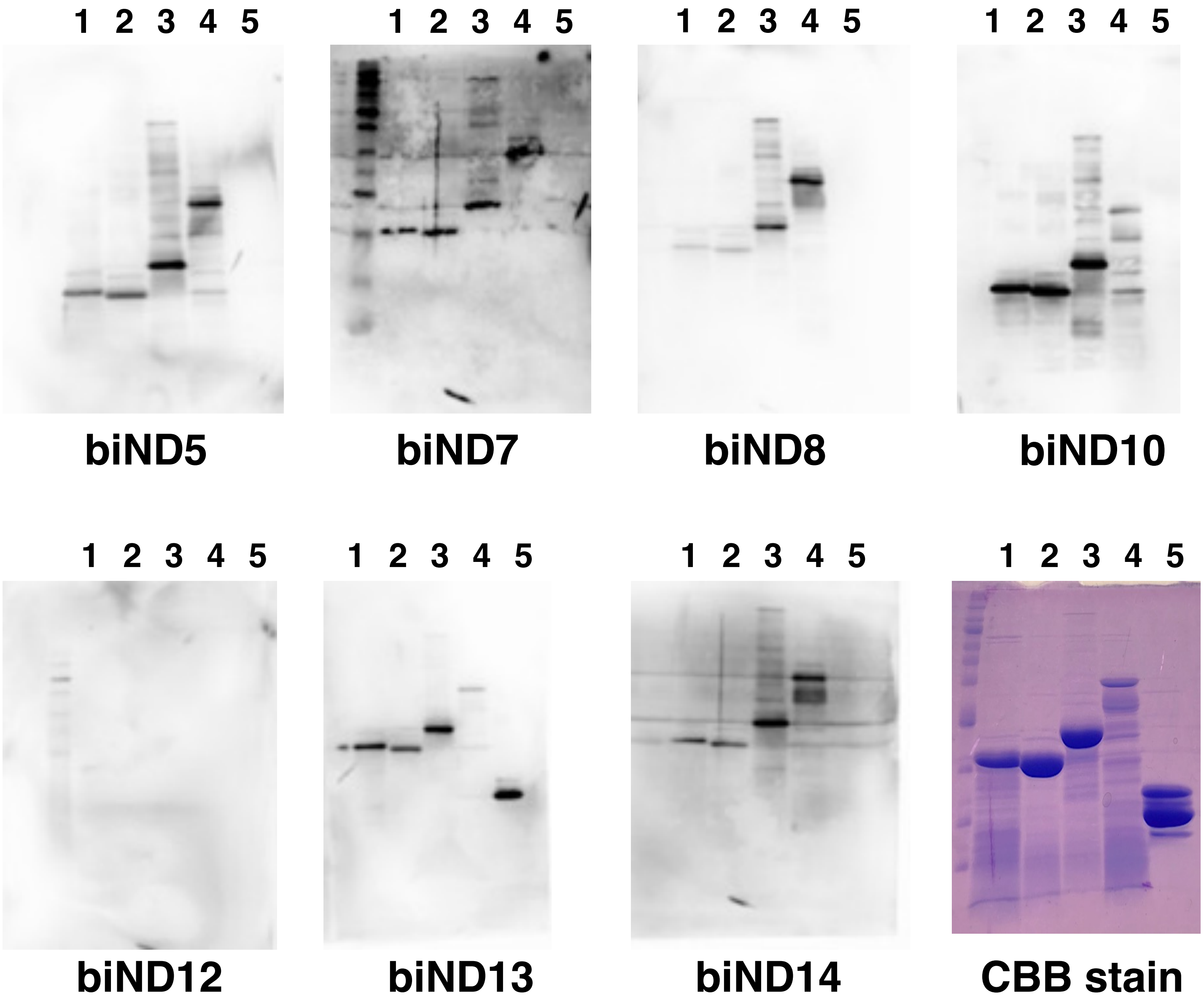

Supplementary Figure S1.

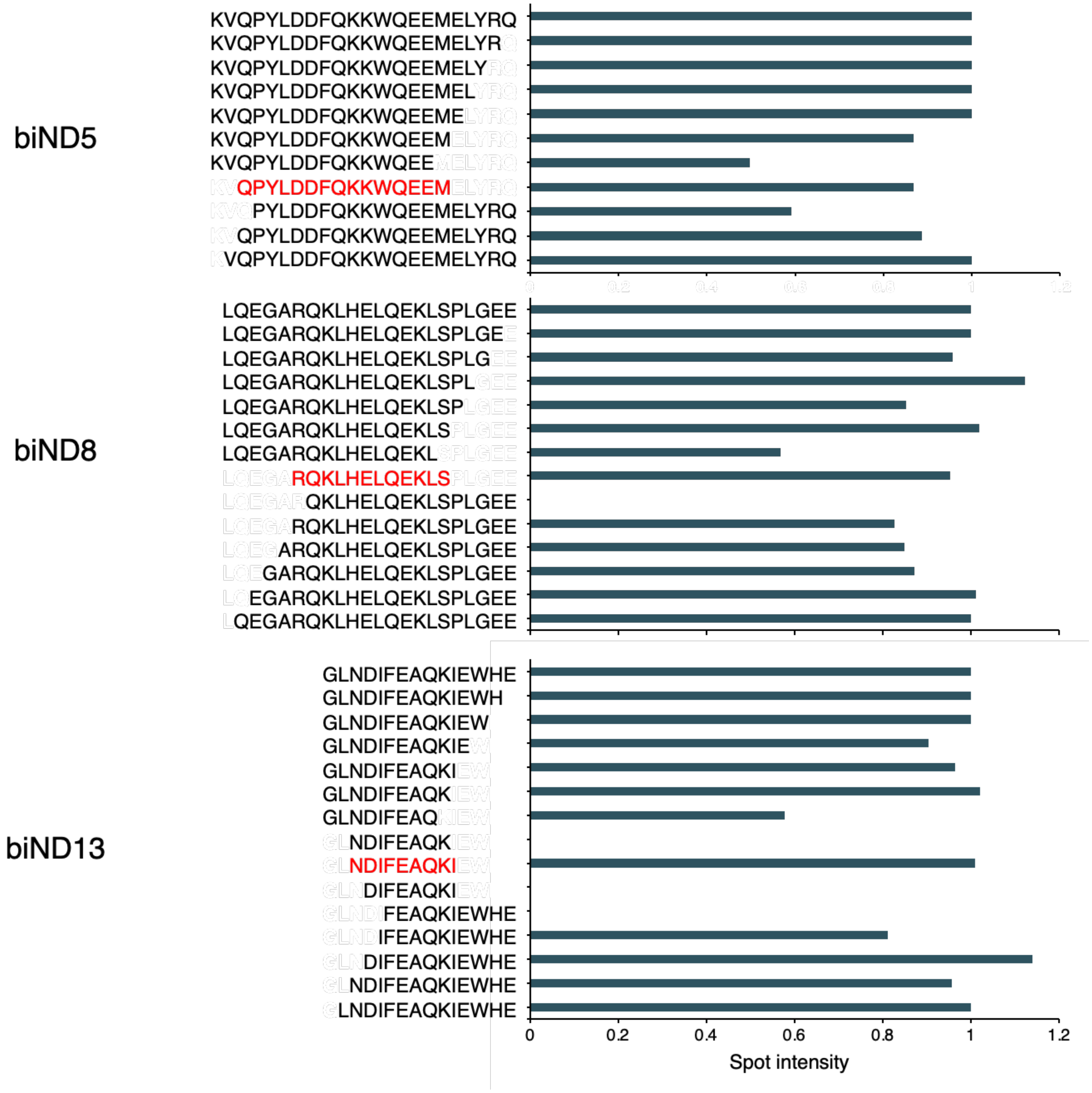

Supplementary Figure S2.

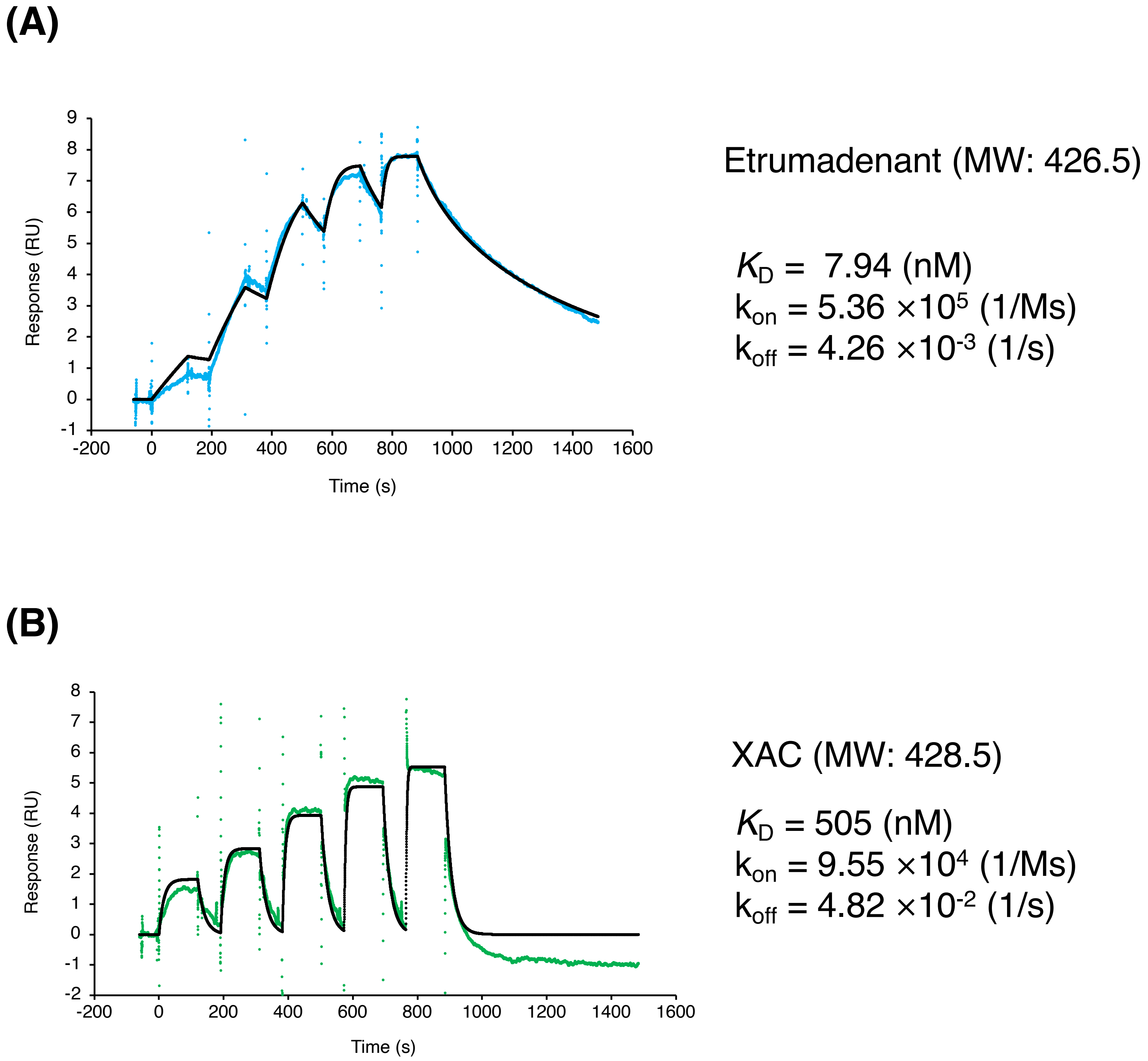

Supplementary Figure S3.

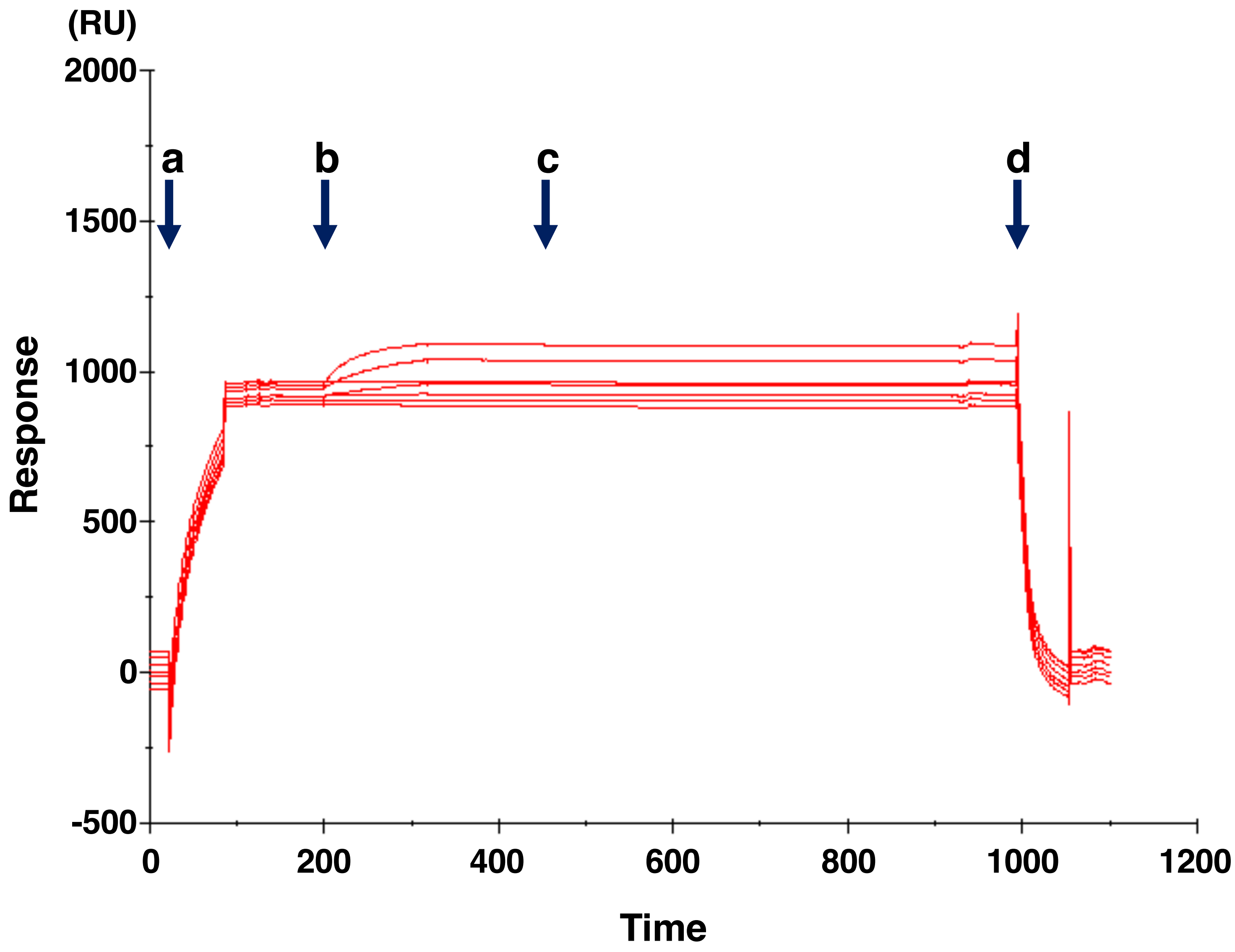

Supplementary Figure S4.

Supplementary Table 1. Affinity against nanodisc (MSP1) and free MSP1.

	nanodisc (MSP1)			Free MSP1		
clones	K_D (nM)	k_{on} (1/Ms)×10^4	k_{off} (1/s)×10^{-4}	K_D (μM)	k_{on} (1/Ms)×10^4	k_{off} (1/s)×10^{-4}
**biND5**	**4.06**	**3.81**	**1.55**	**1.02**	**0.313**	**41.8**
biND7	60.0	3.81	22.8	1.11	1.85	206
biND8	31.4	8.01	25.2	0.297	2.63	78.1
biND10	44.3	2.85	12.6	609	0.00033207	20.2
biND12	92.0	1.97	18.2	15.0	0.00203	3.14
biND13	210	0.750	15.7	30.0	0.00532	16.0
biND14	141	2.14	30.1	38.0	0.00313	11.9

Supplementary Table 2. Affinity against nanodisc (MSP1E3D1) and free MSP1E3D1.

	Nanodisc (MSP1E3D1)			Free MSP1E3D1		
clones	K_D (nM)	k_{on} (1/Ms)×10^4	k_{off} (1/s)×10^{-4}	K_D (μM)	k_{on} (1/Ms)×10^4	k_{off} (1/s)×10^{-4}
**biND5**	**0.784**	**15.1**	**1.17**	**0.198**	**1.53**	**30.1**
biND7	23.5	5.72	13.5	0.216	2.63	56.9
biND8	3.93	33.2	13.0	0.042	1.98	8.42
biND10	2.09	6.43	1.34	2.17	0.372	80.7
biND12	1600	0.0253	4.03	178.0	1.08	19200
biND13	3320	0.0238	7.67	84.2	10.5	88500
biND14	58.6	1.96	11.5	44.3	0.00156	6.93

Supplementary Table 3. Affinity against nanodisc (MSP2N2) and free MSP2N2.

	Nanodisc (MSP2N2)			Free MSP2N2		
clones	K_D (nM)	k_{on} (1/Ms)×10^4	k_{off} (1/s)×10^{-4}	K_D (μM)	k_{on} (1/Ms)×10^4	k_{off} (1/s)×10^{-4}
**biND5**	**2.62**	**9.22**	**2.33**	**0.688**	**0.732**	**49.5**
biND7	47.7	4.24	20.2	0.201	1.76	35.4
biND8	14.1	11.5	16.1	1.040	0.754	78.0
biND10	2.21	5.21	1.15	3.99	0.27507	110
biND12	31.1	4.73	14.7	25.8	0.00233	6.01
biND13	118	2.45	28.9	3.030	0.179	54.2
biND14	33.9	3.95	13.4	96.4	0.00228	22.0
